# Effects of aqueous extract from Baiyedancong-Oolong tea on cytochrome P450 enzymes activities, P-gp and OATs transport abilities and transcription levels in mice

**DOI:** 10.3389/fnut.2023.1136329

**Published:** 2023-05-09

**Authors:** Miaogao Zhang, Zhenguo Qiu

**Affiliations:** College of Light Industry and Food Sciences, Academy of Contemporary Agricultural Engineering Innovations, Zhongkai University of Agriculture and Engineering, Guangzhou, China

**Keywords:** Oolong tea, P-gp, oat, drug metabolism, P450

## Abstract

**Introduction:**

Recent studies have been conducted on its influence on drug metabolism and its potential mechanisms, among which the most studies have been focused on CYP3A enzymes.

**Methods:**

In this study, Baiyedancong Oolong tea (BOT) was processed by freeze- and hot air-drying techniques separately to obtain the aqueous extracts of freeze-and hot-dried BOT (FBOT and HBOT, respectively). High and low doses of FBOT (1463.7 and 292.74 mg/kg/d, respectively) and HBOT (1454.46 mg/kg/d, 290.89, respectively) were administered to mice for 7 days.

**Results:**

Aqueous extracts from BOT simultaneously improved liver CYP3A, CYP2E1, and CYP2C37 activities and weakened the transport ability of P-gp and OATs in a dose-dependent manner, thus affecting multiple links of oral drug metabolism in liver, intestinal absorption and metabolism, and renal excretion. Moreover, aqueous extracts from BOT significantly increased the mRNA expressions of liver CYP3A11 and CYP2C37 as well as intestinal CYP3A11. Decreased transcription levels of MDR1 encoding P-gp in small intestine and renal OAT1 and OAT3, which was in the same direction as the regulation of the above enzyme activities and transport capacities. Besides, the transcription level of liver CYP2E1 was weakened, which was inconsistent with its corresponding enzyme activity, suggesting that the increased CYP2E1 activity may be caused by other mechanisms.

**Conclusion:**

Daily consumption or high dose administration of BOT and its related products may affect drug absorption, metabolisms, and excretion.

## 1. Introduction

As an important representative of Oolong tea in China, Guangdong characteristic Baiyedancong Oolong tea (BOT) was approved by Crop Variety Certification Committee, Ministry of Agriculture as the national tea tree excellent seed in 2002 (1). It has been popularized in Guangdong Province over 200,000 acres and is also distributed in Guangxi, Hainan and other places. Ready-to-drink bottled, or canned tea drinks have been widely integrated into individual’s lives due to their great convenience. Currently, health care tea products are more diversified, such as vitamin complex tablets, various weight loss products. Each tablet contains 50–630 mg of EGCG and caffeine. These products are increasingly being consumed as drug supplements for chronic diseases such as cancer, metabolic and cardiovascular diseases (2).

The improper consumption of tea may bring risks, especially in high and/or long-term doses. Liver poisoning caused by high dosage of green tea polyphenol extract has been reported (3). In the 1980s, Chinese nomadic borderers have paid attention to the fluorosis caused by the long-term drinking of high fluorine old brick tea. In recent years, there has been a great focus on the interaction between tea and drugs (4). According to current reports, tea polyphenols could affect the bioavailability of drugs in various ways, including the direct binding effect with drugs, the activity and expression levels of drug metabolizing enzymes and transporters, thus enhancing or inhibiting the pharmacodynamic effects (5). It has been reported that green tea extract or its main components have significant effects on drug metabolisms, metabolic enzymes and transporter activities when taken in large doses (6). The level of daily consumption could change the blood drug concentration, and the activities of metabolic enzymes and transporters, ultimately affecting the bioavailability and therapeutic effects of drugs (7).

Previous studies mainly focused on the regulation of green tea polyphenol cytochrome P450 (P450), the main enzyme system belongs to phase I metabolic enzymes. The important P450 enzymes in human body mainly include CYP1A2, CYP2A6, CYP2B6, CYP2C8, CYP2C9, CYP2C19, CYP2D6, CYP2E1 and CYP3A4, which are involved in the metabolism of over 90% of clinical drugs and determine their safety and effectiveness (8). Drugs or other exogenous substances can affect the quantities and activities of P450 enzymes, influencing drug metabolism and potentially causing metabolic drug interaction (9). More emphasis has been placed on tea polyphenols’ ability to regulate these enzymes. Drug transporters play an important role in drug absorption, distribution, and excretion and are important for the kinetics of drug metabolism and effects. The effects of certain drugs can be decisive (10). Besides, P-gp can expel drugs from cells, while OATs in the kidney play an important role in the expulsion of organic anions such as uric acid (11), both of which are receiving a lot of attention.

However, the influence of Chinese characteristic Oolong tea (e.g., BOT) on drug metabolizing enzymes and transporters was less involved in previous studies. Compared with green tea, Oolong tea has its unique composition of bioactive components and drinking methods. Even if for the same Oolong tea variety, due to their differences in processing techniques, their compositions and contents of ingredients might be different, and the effects of drug metabolisms *in vivo* may vary (12). In our preliminary study, BOT was processed with freeze-and traditional hot air-drying techniques and then was extracted to obtain the aqueous extracts of FBOT and HBOT, respectively. In this study, the aqueous extracts from FBOT and HBOT were used as the materials to orally administrate to mice for constitutive 7 days separately. The objectives of this study were (1) to exploring their effects on P450 enzymes activities, transport capacities and their corresponding transcription expressions in mice liver and intestine; (2) to evaluate if there is significant difference in these activities and transcription levels between FBOT and HBOT. Our findings may provide a theoretical basis for appropriate consumption of BOT and its related products during medication.

## 2. Materials and methods

### 2.1. Materials

The initial production of BOT was carried out according to the following steps: picking fresh leaves; sunning; cooling; fine manipulation; frying; rolling and drying. Tea samples were rolled and divided into 6 parts and were processed with freeze-and hot air-drying techniques separately. The initial drying temperature during hot air-drying process was 110°C, while the pre-freezing temperature of during freeze-drying process was-20°C. Chromatographic pure methanol and acetonitrile were purchased from Zili Chromatography Co., LTD (Guangzhou, China). NADH^+^ system was bought from Sigma (St. Louis, MO, United States). Erythromycin and aminobiline were purchased from Genview (Houston, TX, USA) and Sinopsin Chemical Reagents Co., LTD. (Shanghai, China), respectively.

### 2.2. Preparation of aqueous extracts from BOT

Freeze-dried and hot air-dried autumn BOT (FBOT and HBOT, respectively) were crushed into tea powders prior to filter through 80-mesh sieve for further analysis. The tea powders (4.375 g) were extracted with 300 ml of boiling ddH_2_O for 10 min. The extract was collected, while the residue was further washed with 100 ml of boiling ddH_2_O for two times, and then the liquid solution was pooled with the extract, which was used for stocking extract. This stocking extract was purified under reduced pressure at 65°C to obtain high concentration extract. Afterwards, the high concentration extract was diluted 5 times to obtain the final BOT working extracts. The high and low concentrations of FBOT (36.59 and 7.32 mg/ml, respectively) and HBOT (36.36 and 7.27 mg/ml, respectively) was obtained in our preliminary study.

### 2.3. Extraction of tea polyphenol

A total of 50 g of tea leaves were extracted with 400 ml of 75% ethanol in room temperature for 4 h. The leaves were separated and extracted with ethanol for 2 time. The ethanol was evaporated in a rotary evaporator under reduced pressure at 40°C. The dried extract was suspended in 500 ml of distilled water and extracted with dichloromethane to remove caffeine. The decaffeinated water solution of tea extract was subjected to an XAD-16 resin column separation, rinsed with 5 volumes of distilled water, and eluted with 100% ethanol. The extract was dried using the rotary evaporator.

### 2.4. Gallic acid equivalent

The assays were performed as reported previously (13) using Folin-Ciocalteau reagent. The absorbance was read at 755 nm in a ThermoMax microplate reader (Molecular Devices). The standard curves were used to convert the average absorbance of each sample into mg/g gallic acid equivalent.

### 2.5. HPLC condition for analysis of amino acids and catechins

A Water Alliance 2,695 HPLC system coupled with a PDA detector was used to analyze the catechins, which was conducted on an Agilent Zorbax SB C18 column with a gradient of acetonitrile and 0.4% phosphoric acid in water. The detection wavelength was 280 nm, while the amino acids were separated on a reversed-phase C18 column (4.6 × 250 mm) at 35°C and monitored at 360 nm. Separation analysis was conducted with a gradient elution of mobile phases at a flow rate of 1 ml/min.

### 2.6. Animal treatment and groups

#### 2.6.1. P450 and P-gp determination and treatment

A total of 96 Specific Pathogen Free (SPF)-grade NIH adult mice (25.0 ± 5.0 g, 48 male and 48 female) were kindly provided by the Laboratory Animal Center of Guangzhou University of Traditional Chinese Medicine (certificate number: SCXK 2013–0020). Mice were fed a standard diet with water *ad libitum* and kept on a 12 h light and dark cycle. Animal welfare and experiments were performed according to institutional guidelines for animal care. The gavage was performed on mice (20 ml/kg) twice a day (8 am and 6 pm) for constitutive 7 days. The mice were randomly divided into 8 groups (*n* = 12), namely control (ddH_2_0), carboxymethylcellulose (CMC, 0.5%, v/v), rifampicin (40 mg/kg, Guangdong Huanan Pharmaceutical Group Co. LTD, Guangzhou, Chian), ketoconazole (1.8 mg/kg, Nanjing Baijingyu Pharmaceutical Co. LTD, Nanjing, China), high and dose of HBOT (HBOT-H and HBOT-L, 1454.46 and 290.89 mg/kg/d, respectively), and high and low dose of FBOT (FBOT-H and FBOT-L, 1463.7 and 292.74 mg/kg/d, respectively). Rifampicin, ketoconazole, and abstention sulfur were all dissolved in 0.5% CMC. On the 8^th^ day, each subject was gavaged in the morning. After 60 min of gavage, all mice were given acetaminophen (APAP, 20 mg/kg) by gavage at the weight of 10 ml/kg mice.

#### 2.6.2. OATs determination and treatment

A total of 84 SPF-grade NIH adult mice (25.0 ± 5.0 g, 42 male and 42 female) were provided by the Laboratory Animal Center of Guangzhou University of Traditional Chinese Medicine. The feeding conditions were the same as above. The gavage was performed on mice (20 ml/kg) twice a day (8 am and 6 pm) for constitutive 7 days. The mice were randomly divided into7 groups (n = 12), namely control (ddH_2_0), CMC (0.5%, v/v), probenecid (50 mg/kg, Sigma, MO, United States), HBOT-H and HBOT-L (1454.46 and 290.89 mg/kg/d, respectively), and FBOT-H and FBOT-L (1463.7 and 292.74 mg/kg/d, respectively). On the 8^th^ day, each subject was gavaged in the morning.

### 2.7. Determination of serum APAP concentration

Refer to the method of Alonso et al. (14). Briefly, blood was collected from the severed head of mice in dry centrifuge tubes after 60 min of APAP administration, and then centrifuged at 2,797.5 × g, 4°C for 5 min after standing for 30 min. The supernatant was collected, among which 0.25 ml of serum was fully mixed with 1 ml of trichloroacetic acid. The mixture was centrifuged at 10,000 × g, 4°C for 5 min, and then 1 ml of supernatant was collected to mix with 0.25 ml of 6 mol/l hydrochloric acid and 0.25 ml of 20% NaNO_2_ solution. The mixture was stand for 5 min for full reaction prior to slow addition of 0.5 ml of 15% sulfionic acid solution, using ultrasound to ensure no bubbles were generated. Afterwards, 1 ml of 20% NaOH solution was added and left at room temperature for 20 min. The absorbance was measured at 430 nm. The mixture without APAP was used as control. When the linear range of APAP was between 0.5 and 50 ug/mL, the within-day precision was between1.35 and 2.12%, while the inter-day precision was between 2.78 and 3.58%. The reproducibility was 98–113%.

### 2.8. Extraction of liver and intestinal microsomes

Refer to Hatley et al. (15) for the separation method, liver and intestinal microsomes (LM and IM, respectively) were separated by calcium precipitation differential centrifugation assay. Liver and small intestine were extracted with phosphate balanced solution (PBS, pH = 7.4) at 0°C using tissue homogenizer at a speed of 142 × g for 5 times in a total of 30 s. Thereafter, the mixture was centrifugated at 9,000 × g for 20 min, 50,000 × g for 30 min, and 50,000 × g for 30 min in turn. The precipitates were collected and mixed with the same amount of PBS solution. The LM and IM were stored at-80°C. The protein concentration of LM and IM was determined by Bradford protein concentration assay kit (Jiemei Gene Medicine Technology Co., LTD, Shanghai, China).

### 2.9. Determination of CYP3A activity in mice LM and IM

LM and IM samples (0.5 ml) containing 1 mg/ml of protein was mixed with 0.3 ml of 0.02 mol/l HEPES buffer (pH 7.4, Dingguo Changsheng Biotechnology Co., LTD, Beijing, China), and 0.1 ml of 0.4 mmol/l erythromycin or 86.4 mmol/l aminopyrine in a 37°C water bath for 3 min. NADPH-generating system (2 mmol/l NADP^+^, 20 mmol/l G-6-P, 20 mmol/l MgCl_2_ and 2 U/ml G-6-PDH) was added into the mixture to incubate at 37°C for 60 min. The reaction was terminated by adding 0.5 ml of 30% trichloroacetic acid, followed by centrifugation (20,000 × g, 0°C for 10 min). Supernatant (1.0 ml) was collected to mix with 1 ml of NASH reagent in a 60°C water bath for 10 min prior to standing at room temperature for 20 min. The absorbance was determined at 412 nm wavelength (16). The enzyme activity was expressed as nmol product/min/nmol P450. The standard curve of formaldehyde was as following: *Y* = 0.1945X + 0.0157, *r* = 0.9988, *n* = 5 (linear range: 1.5–15 nmol), *Y* = 0.1947X + 0.0156, *r* = 0.9988, *n* = 5 (linear range: 0.02–0.4 nmol) and *Y* = 0.1945X + 0.0157, *r* = 0.9988, *n* = 5 (linear range: 0.05–0.5 nmol), corresponding to the determination of liver CYP3A activity in mice using erythromycin, aminopyrazoline and erythromycin as substrates, respectively.

### 2.10. Determination of CYP2E1 activity in mice LM

LM suspension (0.9 ml) was mixed with 0.05 ml of 0.1 mol/l aniline solution in a 37°C water bath for 3 min. Hydroxyl isopropyl benzene peroxide solution (0.05 ml) was added to the mixture and continued shaking for 3 min. Thereafter, 35% trichloroacetic acid (0.4 ml) was added to centrifuge at 20,000 × g, 0°C for 10 min. Supernatant (1 ml) was collected to mix with 0.5 ml of 1 mol/l sodium carbonate solution. Afterwards, 0.5 ml of 2% phenol reagent was added to react at room temperature for 30 min. The absorbance was determined at 630 nm wavelength (17). The content of p-aminophenol was calculated. The CYP2E1 activity was expressed as nmol product/min/nmol P450. The standard curve obtained was *Y* = 0.005X - 0.087, *R* = 0.997, *n* = 5 (linear range: 12.5–125 nmol).

### 2.11. Determination of CYP2C37 activity in mice LM

Chromatographic column conditions: Agilent ZORBAX SB-C18 reversed-phase column (150 mm × 4.6 mm, 5 μm); mobile phase: acetonitrile-0.01 mol /L ammonium acetate (48:52 (v/v), pH = 3.6); flow rate: 1.00 ml/min; column temperature: 30°C; detection wavelength: 280 nm. The enzyme reaction system includes 20 μl of 80 μmol/l diclofenac solution, 50 μl of 1 mg/ml LM protein solution, 30 μl of NADPH generation system, 80 μl of potassium phosphate buffer (pH = 7.4). This enzyme system was pre-incubated in a 37°C water bath for 3 min prior to adding 20 μl of β-NADP^+^ to start the reaction, and then incubated for 15 min, followed by adding 180 μl of acetonitrile to stop the reaction. Afterwards, 20 μl of internal standard solution was added to centrifugate at 4°C at 12,000 × g for 15 min. The supernatant (20 μl) was collected for HPLC analysis (*n* = 6). Gradients doses of 0.5, 1, 2.5, 5, 7.5, 10, 15, 20 μmol/l of 4 ‘-hydroxydiclofenac standard enzyme incubation system was analyzed by HPLC. The standard curve was drawn with the dose of 4 ‘-hydroxydiclofenac [C (μmol/L), X] as the horizontal coordinate and the peak area ratio of 4′ -hydroxydiclofenac to internal standard (A1/A2, Y) as the vertical coordinate. The standard curve obtained was *Y* = −0.7618X + 1.8178, *R* = 0.9997, *n* = 5 (linear range: 0.5–20 μmol/l). The detection limit (LOD) was 0.25 μmol/l (S/N ≥ 3, *n* = 5), and the RSD was less than 1.15% for 5 parallel measurements (18).

### 2.12. Extraction of total RNA

Total RNA was extracted from mice liver, small intestine, and left kidney accordance with the instructions of RNA extraction kit (TaKaRa, Japan). Absorbance was measured at 260 and 280 nm by ultraviolet spectrophotometry. Samples with A260nm/A280nm between 1.8–2.2 were used for real time quantitative PCR (RT-PCR) analysis.

### 2.13. Determination of liver and intestine CYP3A11, liver CYP2E1, liver CYP2C37, intestinal MDR1, renal OAT1 and OAT3 mRNA levels in mice

The reverse transcription was performed according to the instructions of the PrimeScript™ RT assay kit (Takara, Tokyo, Japan) by using ABI7500 real-time fluorescent quantitative PCR equipment. The reaction conditions were: 42°C for 15 min; 85°C for 31 s for one cycle. The reaction system included 2 μl of total RNA, 2.5 μl of PrimeScript®Buffer (5x), 1 μl of Oligo dT Primer (50 μmol/l), 3 μl of Random 6mers (100 μmol/l), and using ddH_2_O (containing no RNase) to make up to 20 μl. PCR reaction was performed using the SYBR®Premix Ex Taq™II assay kit (Takara, Tokyo, Japan) in a 20 μl system containing 10 μl of SYBR® Premix Ex Taq™II, 0.8 μl of forward and reverse primers (Shanghai Sangong Biological Engineering Co., LTD, Shanghai, China) separately, 0.4 μl of ROX Rreference DyeII, 2 μl of cDNA sample, and using DEPC water make up to 20 μl. PCR reaction were as follows: pre-degeneration at 95°C for 30 s, and then entered the cycle stage (40 cycles), each cycle included 95°C denatured for 15 s, annealing at 56°C for 30 s, and extension at 72°C for 31 s. GAPDH was applied as internal reference, the relative expression levels of target genes were calculated by 2^-△△Ct^. The Primes sequences were list in Table 1.

**Table 1:** Prime sequences for RT-PCR.

Gene	Forward prime(5′ → 3′)	Reverse prime(5′ → 3′)
CYP3A11	CTCAATGGTGTGTATATCCCC	CCGATGTTCTTAGACACTGCC
CYP2E1	CACCGTTGCCTTGCTTGTCTG	CTCATGAGCTCCAGACACTTC
CYP2C37	CTGCATGACAGCACGGAGTT	GTGGCCAGGGTCAAATTTCTC
MDR1	CCCATCATTGCAATAGCAGG	GTTCAAACTTCTGCTCCTGA
OAT1	ATGCCTATCCACACCCGTGC	GGCAAAGCTAGTGGCAAACC
OAT3	CAGTCTTCATGGCAGGTATA	CTGTAGCCAGCGCCACTGAG
GAPDH	GGTGAAGGTCGGTGTGAACG	CTCGCTCCTGGAAGATGGTG-

### 2.14. Determination of transporter P-gp coupled ATPase activity

The transporter P-gp coupled ATPase activity was measured according to the instructions of the ultramicro ATPase kit (Abcam, Cambridge, FC, United States).

### 2.15. *In vitro* PAH uptake in renal sections

The mice were sacrificed 45 min after the last gavage. Both sides of kidney were immediately removed, among which the right kidney was stored at-80°C for mRNA determination, while the left kidney was cut into 2 parts on average along the long axis from the renal hilum, and each part was equally cut into 3 strips along the long axis and was further cut into 3 pieces equally. After being rinsed with cold PBS, the kidney was dried with filter paper and put into a 12-well culture plate containing 1 ml of PBS with sufficient oxygen. A slice of kidney was put into a well containing 2 mmol/l PAH and incubated in a 37°C, 5% CO_2_ incubator for 20 min, and shook for 5 s every 5 min. 10% trichloroacetic acid solution (250 μl) was added to stop the reaction. The incubated renal tissues were homogenized with 5-time of phosphoric acid buffer (pH = 7.4) (20 s × 5 times, 10 s at each interval). After centrifugation at 2,000 × g for 30 min, the supernatant was collected to determine the protein concentration by Folin phenol method. The PAH in supernatant was determined according to a previous study (19). The PAH standard curve obtained is *y* = 0.3937x-0.0025, *R*^2^ = 0.9986, *n* = 10.

### 2.16. Statistical analysis

SPSS13.0 statistical software was applied. The results were expressed as mean ± standard error (SE), and inter-group *t* test was conducted to analyze the differences between groups. *p* < 0.05 and *p* < 0.01 were considered statistically significant.

## 3. Results

### 3.1. The main compositions in FBOT and HBOT

Tea aqueous extract is a general term for tea that is soluble in hot water, containing tea polyphenols and amino acids. Tea with high content of tea aqueous extract always exhibits strong taste. In our preliminary study, BOT obtained from different seasons (spring, summer, autumn and autumn) and processed by different techniques (vacuum freeze-drying, vacuum cryodrying and heat-drying) were employed to compare their sensory characteristics, the content of aqueous extract and the main bioactive compounds in BOT (data was not shown). The result showed that the autumn FBOT achieved the highest score in sensory assessment, and its tea aqueous extract content (41.82%) was also much higher than other BOT. Tea polyphenols account for 18–36% of tea dry weight and are one of the main substances that produce bitter taste in tea. Herein, TP content in the autumn HBOT and FBOT was 24.13 and 25.76%, respectively, which was the highest among the autumn BOT processed by different techniques. The amino acids content in autumn HBOT and FBOT account for 2.51 and 3.47%, respectively, which are important flavors in tea and play an active role in the freshness and flavor of tea soup. Freeze-drying technique has less effect on catechins contents. EGCG, EGC, ECG, and EC are four main monomers of catechins in Oolong tea. Herein, the main total catechins contents in autumn HBOT and FBOT was 183.23 and 209.38%, respectively, which had a significant difference between these two teas. This suggested that vacuum freeze-drying may retain more EGCG, ECG, EGC, and EC than other drying methods (Table 2).

**Table 2:** Main compositions in HBOT and FBOT.

Compositions	HBOT	FBOT
Aqueous extracts (%)	41.56 ± 1.02a	41.82 ± 1.54a
Total polyphenols (%)	24.13 ± 1.21b	25.76 ± 1.17a
Amino acids (%)	2.51 ± 0.05b	3.47 ± 0.07a
Catechins (mg/g)	183.23 ± 3.02b	209.38 ± 2.24a

Values are mean ± standard deviation (SD, *n* = 3). Values with different letters in the same row represent there is significant difference between HBOT and FBOT (*p* < 0.05). HBOT = hot dried Baiyedancong Oolong tea; FBOT = freeze-dried Baiyedancong Oolong tea.

### 3.2. The effects of aqueous extracts of FBOT and HBOT on LM CYP3A activity and mRNA expression in mice

**Figure 1:**
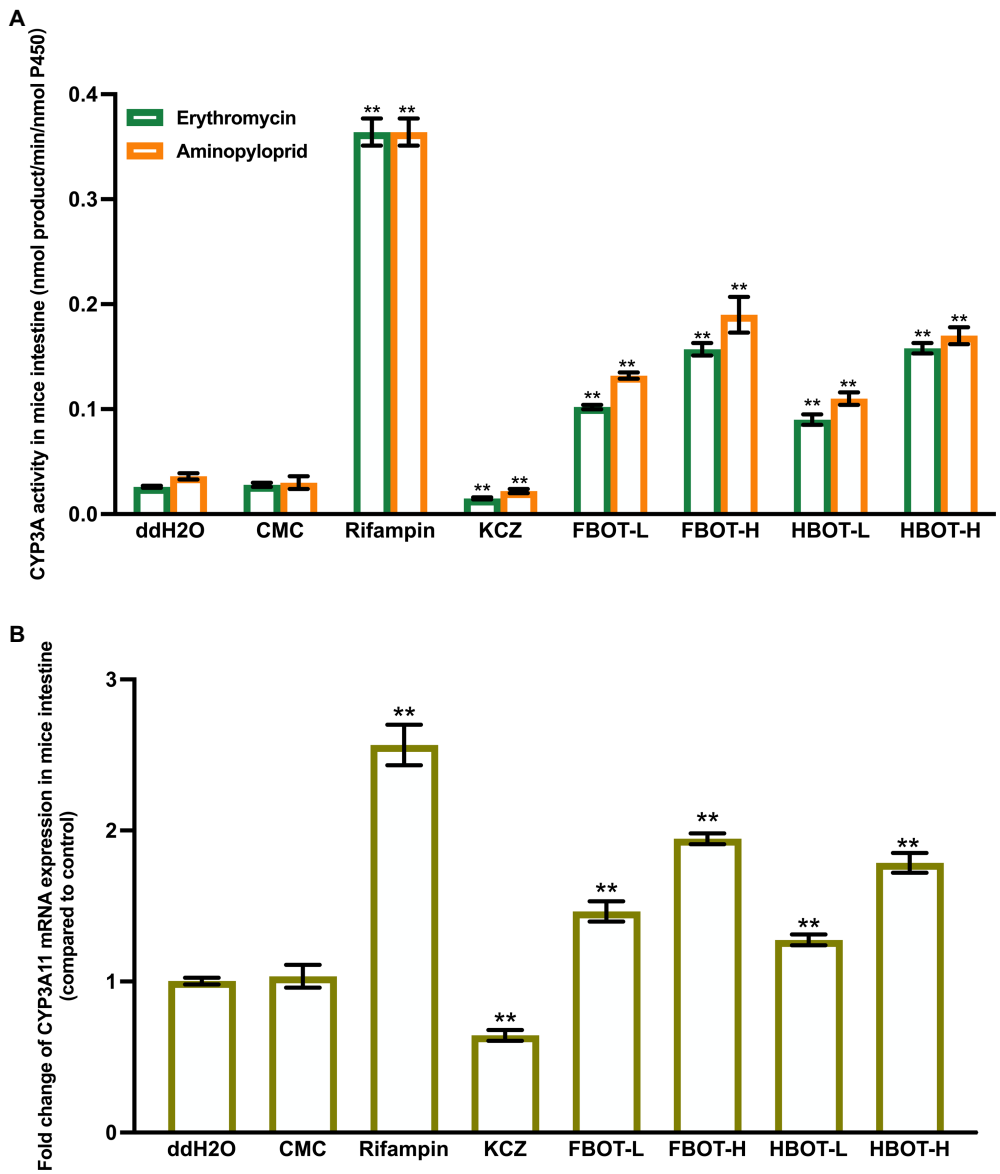
The activities of CYP3A **(A)** and mRNA expression of CYP3A11 **(B)** in mice liver. Values are mean ± SD (*n* = 3). Bars with uppercase letters (aminopyrine as substrate) and lowercase letters (erythromycin as substrate) represent a significant difference between each group (*p* < 0.05). FBOT-L and FBOT-H mean low and high dose of freeze-drying Baiyedancong Oolong tea, respectively; HBOT-L and HBOT-H mean low and high dose of hot air-drying Baiyedancong Oolong tea, respectively.

CYP3A is one of the key isoenzymes involved in the metabolism of clinical drugs. Herein, CYP3A activity was determined with two substrates, erythromycin and aminopyloprid separately, which displayed similar results as shown in Figure 1A. There was no significant difference in LM CYP3A activity between CMC and control groups, suggesting that CMC did not influence the LM CYP3A activity. LM CYP3A activity in rifampicin-induced mice was 6.73 times higher than that in the control group (*p* < 0.01), while the CYP3A activity in ketoconazole-treated mice was significantly lower than that in the control group (78% of the control group). Compared with the control group, LM CYP3A activity of mice treated with FBOT and HBOT was significantly increased in a dose-dependent manner, among which the CYP3A activity in FBOT-H treated mice reached to the level of rifampicin-induced mice. There was no significant difference in LM CYP3A activity between FBOT and HBOT-treated mice (*p* > 0.05). Despite the difference in chemical compositions between FBOT and HBOT, their effects on inducing LM CYP3A activity in mice showed no obvious difference. Herein, both high and low dose of FBOT and HBOT significantly increased the activity of LM CYP3A, which may enhance the liver metabolism of substrate drugs and reduce the amount of substrate drugs entering the large circulation in the body.

In order to study whether the increased liver CYP3A activity is caused by the transcription of CYP3A11 encoding gene, CYP3A11 mRNA level in mice liver was determined by RT-PCR assay. The results are shown in Figure 1B. There was no significant difference in the mRNA expression of CYP3A11 between the control and CMC group (*p* > 0.05). Rifampicin significantly increased the mRNA expression level of CYP3A11 in mice liver, which was 2.23 times that of the control group, while ketoconazole decreased its expression, only 70% of the control group (*p* < 0.05). The LM CYP3A11 mRNA expression in mice treated with high and low doses of FBOT and HBOT increased dramatically, which was 1.63, 1.27, 1.62 and 1.19 times that of the control group, respectively. At the same dose level, no obvious difference was found between FBOT and HBOT.

Collectively, the increased LM CYP3A11 mRNA expression in mice treated with FBOT and HBOT aqueous extracts is consistent with its CYP3A11 activity in mice liver. In addition, this effect of BOT-H aqueous extracts and rifampicin was in a comparable level. However, the induction of CYP3A11 mRNA expression is significantly lower than rifampicin. This revealed that in addition to inducing the CYP3A11 mRNA expression, the BOT aqueous extracts may also improve the activity through other ways, such as improving the translation level, modifying post-translation, and stabilizing enzyme quantities.

### 3.3. The effects of aqueous extracts of FBOT and HBOT on LM CYP2E1 activity and mRNA expression in mice

**Figure 2:**
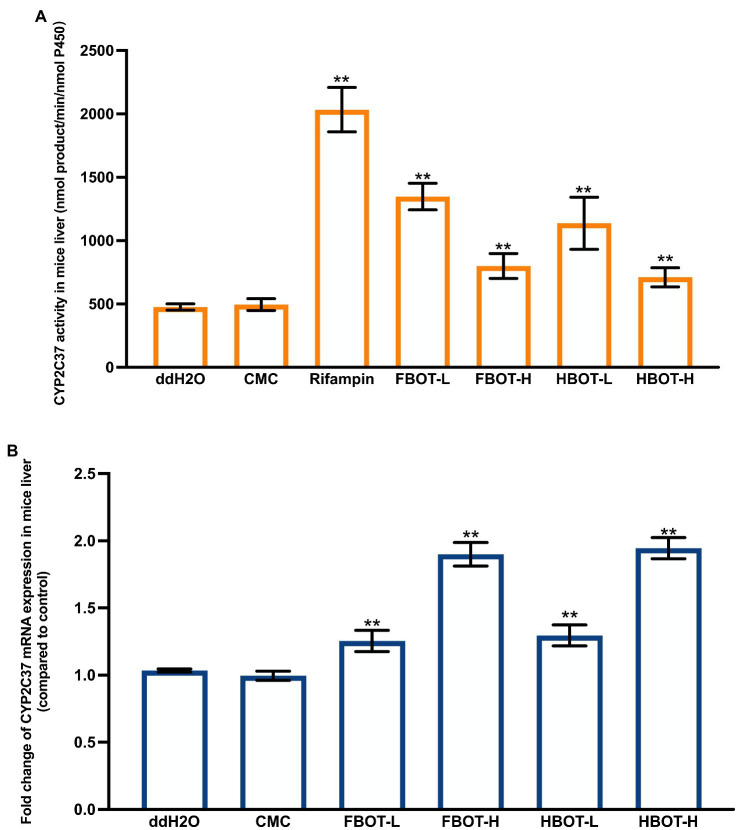
The activities of CYP2E1 **(A)** and mRNA expression of CYP2E1 **(B)** in mice liver. Values are mean ± SD (*n* = 3). Bars with different letters represent a significant difference between each group (*p* < 0.05). FBOT-L and FBOT-H mean low and high dose of freeze-drying Baiyedancong Oolong tea, respectively; HBOT-L and HBOT-H mean low and high dose of hot air-drying Baiyedancong Oolong tea, respectively.

The LM CYP2E1 activity in mice with different treatments have shown in Figure 2A. Compared with the control group, LM CYP2E1 activity in CMC-treated mice did not change significantly, while it was decreased in alcohol abstinence sulfur-treated mice, with only 67% of the control group. Both FBOT and HBOT-treated mice showed stronger LM CYP2E1 activities as comparing to the control group (*p* < 0.05). The CYP2E1 activity of FBOT-L and FBOT-H groups were 6.79 and 2.85 times of that the control group, respectively, while it was 6.81 and 2.82 times of that the control group in HBOT-L and HBOT-H treated mice, respectively. However, no significant difference observed in LM CYP2E1 activity between mice treated with FBOT and HBOT (*p* > 0.05).

The mRNA expression levels of CYP2E1 in mice liver administrated with different treatments have shown in Figure 2B. Generally, CYP2E1 mRNA level in the control and CMC groups had no significant difference (*p* > 0.05). Compared with the control group, abstinence sulfur reduced CYP2E1 mRNA expression by half. Similarly, CYP2E1 mRNA expression in mice treated with high and low dose of FBOT and HBOT were also significantly downregulated (*p* < 0.05), which was 61, 77, 62 and 78% of the control group, respectively. Moreover, this mRNA expression was significantly different between high and low dose treatments. However, at the same dose level, no obvious difference was observed between FBOT and HBOT. Herein, CYP2E1 mRNA expression was inconsistent with the reaction of CYP2E1 activity to BOT. The mRNA expression of CYP2E1 treated with BOT was significantly decreased and the transcription was inhibited, while the activity of CYP2E1 was increased, which suggested that this increased enzyme activity should not be resulted from the upregulated CYP2E1 mRNA expression but might be related to such factors as the amount of stable enzyme.

### 3.4. The effects of aqueous extracts of FBOT and HBOT on LM CYP2C37 activity and mRNA level in mice

**Figure 3:**
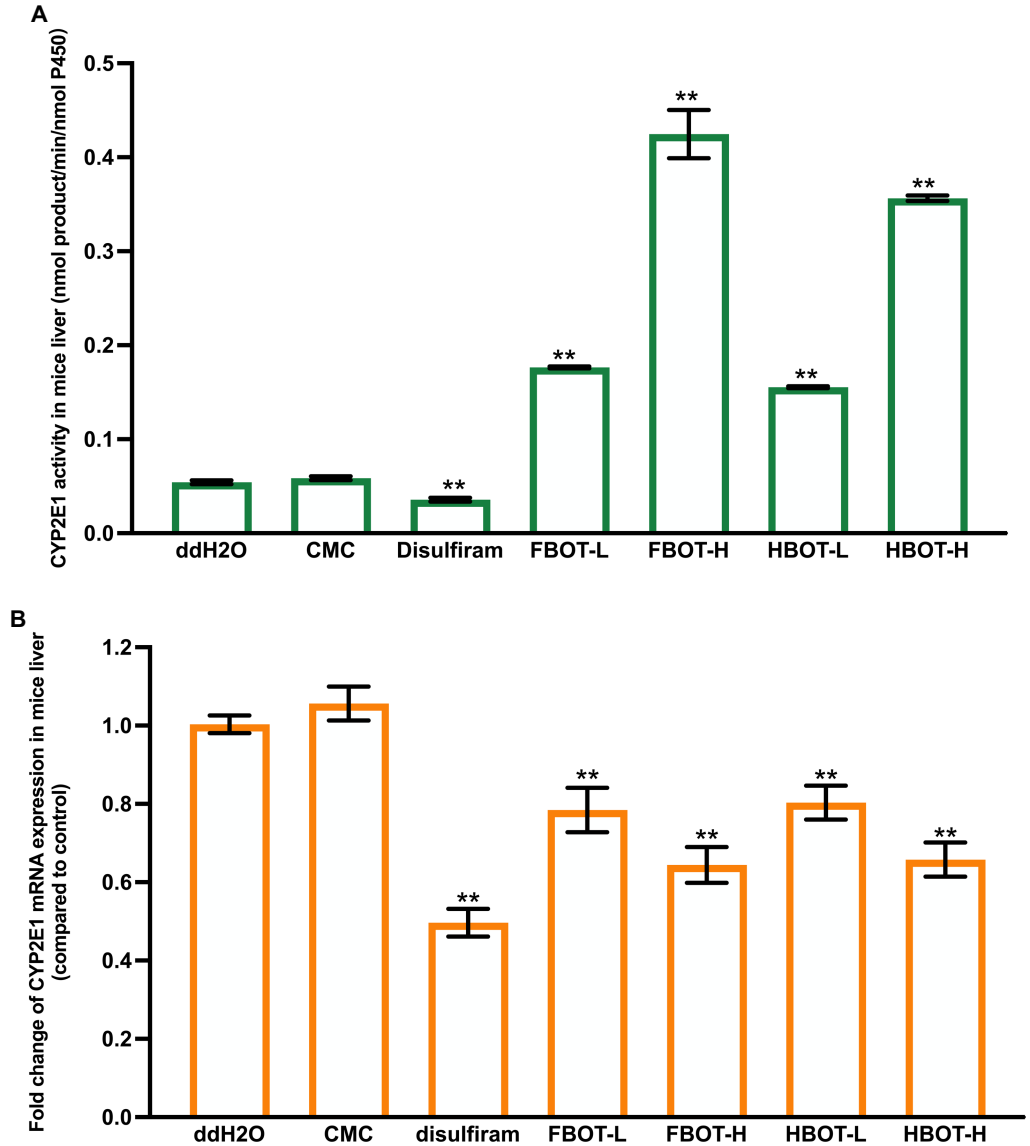
The activities of CYP2C37 **(A)** and mRNA expression of CYP2C37 **(B)** in mice liver. Values are mean ± SD (*n* = 3). Bars with different letters represent a significant difference between each group (*p* < 0.05). FBOT-L and FBOT-H mean low and high dose of freeze-drying Baiyedancong Oolong tea, respectively; HBOT-L and HBOT-H mean low and high dose of hot air-drying Baiyedancong Oolong tea, respectively.

Figure 3A displays the LM CYP2C37 activity of mice with different treatments. Compared with the control group, the LM CYP2C37 activity of CMC-treated mice had no significant change, suggesting that CMC did not influence the CYP2C37 activity. Rifampicin significantly increased the activity of liver CYP2C37 in mice, which was 5.33 times higher than that in the control group (*p* < 0.05). BOT extracts enhanced CYP2C37 activity in mice in a dose-dependent manner. CYP2C37 activity of FBOT-L and FBOT-H-treated mice were 2.65 and 1.67 times higher than that of the control group, respectively, while it was 2.63 and 1.62 times higher, respectively, in HBOT-L and HBOT-H mice, than that of the control group.

No significant difference was observed in the mRNA expression of CYP2C37 between the control and CMC group (Figure 3B), indicating that CMC had no influence on CYP2C37 mRNA level. Rifampicin increased the expression of liver CYP2C37 mRNA expression, up to 2.23 times that of the control group. Similarly, this expression was also increased in mice treated with both high and low doses of FBO and HBOT (*p* < 0.05), which was 1.77, 1.23, 1.82 and 1.25 times that of the control group, respectively. Moreover, the difference between the low and high dose was extremely significant. There was no significant difference in the CYP2C37 mRNA expression between FBOT and HBOT. Collectively, the aqueous extracts from BOT have consistent regulation on CYP2C37 mRNA level and its enzyme activity, indicating that the increased CYP2C37 enzyme activity might be closely related to the upregulation of CYP2C37 mRNA expression.

Comprehensive comparison of the activities and mRNA expressions of CYP3A, CYP2E1 and CYP2C37 in LM revealed that high and low doses of FBOT and HBOT could significantly improve the activities of these three enzymes and its mRNA expressions. Besides, there was no significant difference between FBOT and HBOT when treated with the same dose.

### 3.5. The effects of aqueous extracts of FBOT and HBOT on IM CYP3A activity and mRNA expression in mice

**Figure 4:**
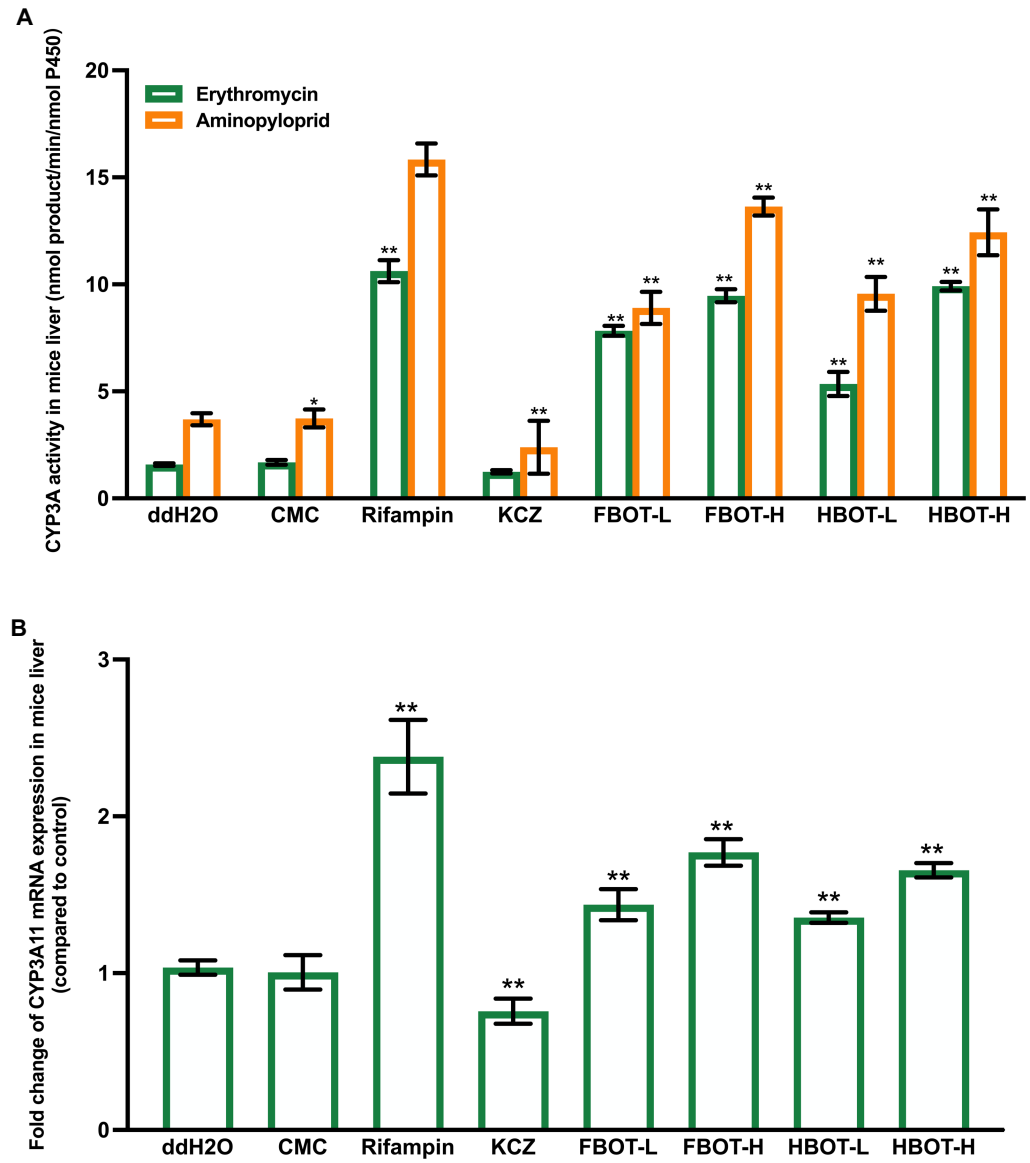
The activities of CYP3A **(A)** and mRNA expression of CYP3A11 **(B)** in mice intestine. Values are mean ± SD (*n* = 3). Bars with uppercase letters (aminopyrine as substrate) and lowercase letters (erythromycin as substrate) represent a significant difference between each group (*p* < 0.05). FBOT-L and FBOT-H mean low and high dose of freeze-drying Baiyedancong Oolong tea, respectively; HBOT-L and HBOT-H mean low and high dose of hot air-drying Baiyedancong Oolong tea, respectively.

According to Figure 4A, The IM CYP3A activity with erythromycin as the substrate was as similar with that of aminopyriprid. CMC had no effect on the IM CYP3A activity, while rifampicin significantly increased the IM CYP3A activity, which was 13.84 times higher that of the control group (*p* < 0.01). Ketoconazole reduced the IM CYP3A activity obviously, only 55% of that in the control group. Moreover, high and low dose of FBOT and HBOT extract significantly improved the IM CYP3A activity in a dose-dependent manner (*p* < 0.01), with 5.96, 3.87, 6.01 and 3.73 times higher that of the control group, respectively. However, at the same dose level, there was no significant difference in the activity of CYP3A between FBOT and HBOT. In addition, the enhancement degree of rifampicin on IM CYP3A activity was significantly higher than that of the LM CYP3A activity. These observations suggest that both FBOT and HBOT extracts were likely to enhance intestinal metabolism of substrate drugs and reduce the amount of substrate drugs that could be absorbed through the intestine.

The mRNA expression of CYP3A11 in the control and CMC group had no difference (Figure 4B). Rifampicin significantly increased the small intestine CYP3A11 mRNA expression level, which was 2.56 times that of the control group, while ketoconazole decreased its expression, only 63% of the control group (*p* < 0.05). The mRNA level of CYP3A11 in small intestine of mice treated with high and low doses of FBOT and HBOT was significantly improved, which was 1.94, 1.32, 1.85 and 1.23 times that of the control group, respectively. The regulation of CYP3A11mRNA and its enzyme activity by aqueous extracts from BOT is consistent. The observation indicated that the increased CYP3A enzyme activity was related to the upregulation of CYP3A11 mRNA expression.

### 3.6. The effects of FBOT and HBOT aqueous extracts on IM ATPase activity and MDR1 mRNA expression in mice

**Figure 5:**
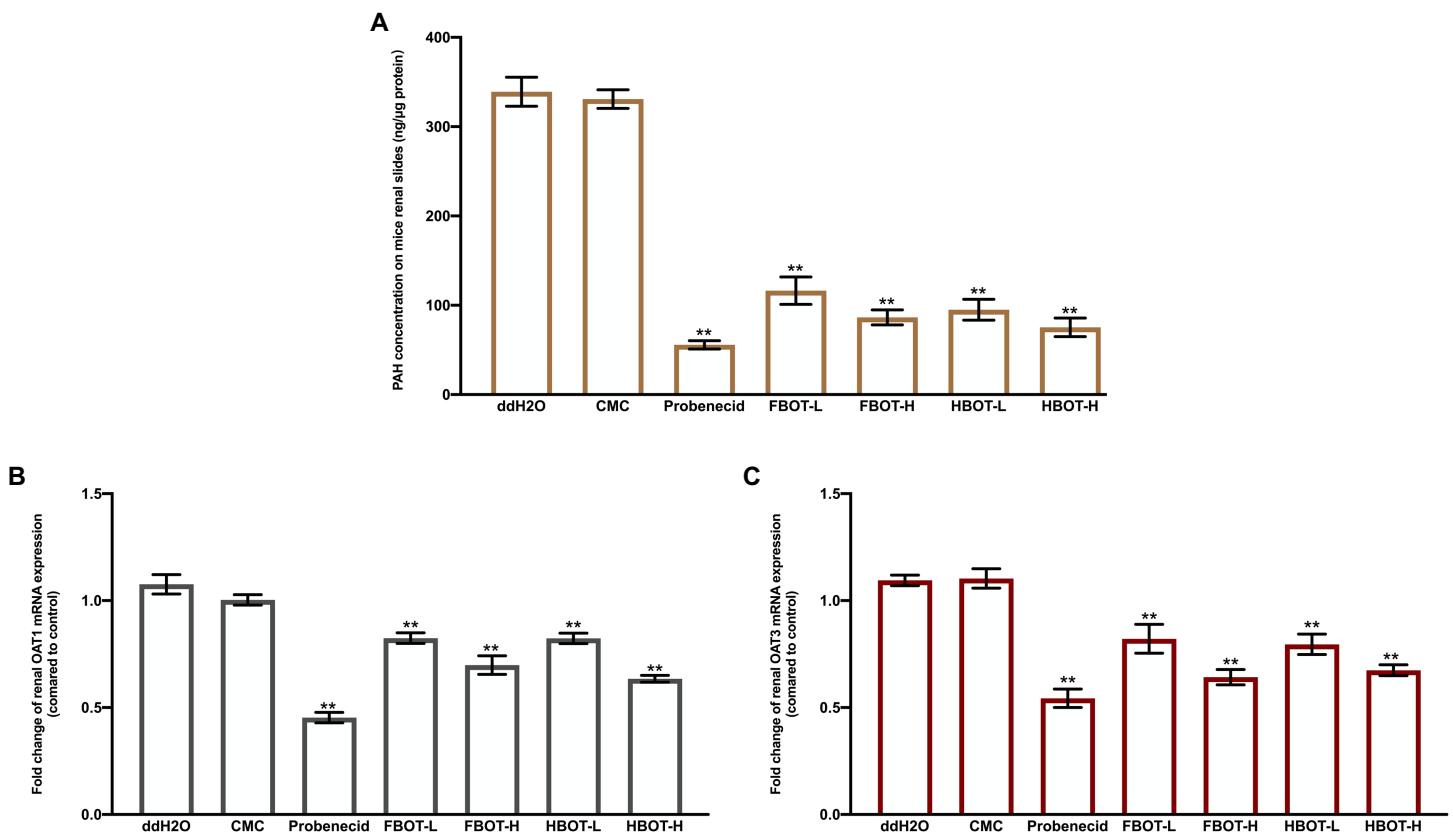
The ATPase activity in mice liver **(A)**, serum APAP concentration in mice **(B)** and MDR1 mRNA expression **(C)**. Values are mean ± SD (*n* = 3). Bars with different letters represent a significant difference between each group (*p* < 0.05). FBOT-L and FBOT-H mean low and high dose of freeze-drying Baiyedancong Oolong tea, respectively; HBOT-L and HBOT-H mean low and high dose of hot air-drying Baiyedancong Oolong tea, respectively.

No direct indicators have been reported to detect P-gp function *in vivo* till now. Currently, the ATPase activity or drug accumulative concentration that powers P-gp is commonly applied to indicate the P-gp function. As shown in Figure 5A, compared with the control group, the P-gp coupled ATPase activity in IM of CMC-treated mice did not change significantly, while rifampicin increased the IM ATPase activity obviously, which was 1.60 times higher than that of the control group (*p* < 0.05). Besides, ketoconazole reduced the IM ATPase activity, which was 0.51 times that of the control group (*p* < 0.05). High and low doses of FBOT and HBOT extracts significantly increased the IM ATPase activities, which was only 49, 66, 57 and 72% of the control group, respectively. However, at the same dose level, there was no significant difference in ATPase activity between FBOT and HBOT. As ATPase is a key enzyme providing energy for P-gp, herein, both FBOT and HBOT significantly reduced the activity of ATPase in the IM of mice, which weakened the transport function of P-gp in the intestine, thereby reducing the outflow of substrate drugs absorbed into the blood and increasing the amount of substrate drugs absorbed through the intestine.

### 3.7. Effects of FBOT and HBOT aqueous extracts on serum APAP concentration and MDR1 mRNA expression in mice

Furthermore, the multipoint serum concentration of APAP, a typical P-gp substrate drug, was used to infer if the transport capacity of P-gp was regulated, and the IM ATPase activity was confirmed. One hour of the last gavage Serum APAP concentration was measured at five time points of 30, 45, 60, 75 and 90 min. The results are shown in Figure 5B. Generally, APAP concentration reached the peak at 75 min. No significant difference was observed between the control and CMC group (*p* > 0.05), indicating that CMC had no significant influence on serum APAP concentration of mice. Ketoconazole, a P-gp inhibitor, significantly increased serum APAP concentration, suggesting that ketoconazole reduced the efflux effect of P-gp on substrate APAP and increased the amount of its absorption through the intestine. Besides, the powerful inducer rifampicin obviously reduced the serum APAP concentration in mice. This revealed that rifampicin could enhanced the efflux effect of transporter P-gp on the substrate APAP and reduced the amount of substrate APAP absorbed through the intestine. Moreover, APAP concentration in FBOT-and HBOT-treated mice was higher than that in the control group, while it was lower than that in ketoconazole group. The peak value of FBOT-H and HBOT-H group was 22.69 and 22.22 μg/ml, respectively, while the peak value of FBOT-L and HBOT-L was 18.30 and 17.17 μg/ml, respectively.

The mRNA expression of small intestine MDR1 in mice was determined by RT-PCR assay to investigate if the reduced transport capacity of P-gp was resulted from the weakened transcription of encoding gene MDR1. The results are displayed in Figure 5C. Herein, the MDR1 mRNA expression showed a similar pattern with the serum APAP concentration. Both ketoconazole and BOT significantly decreased the expression of MDR1 mRNA expression in small intestine compared to the control group (*p* < 0.05). Combined with mRNA expression serum APAP concentration analysis, the regulation of MDR1 mRNA expression and P-gp capacity was consistent, which were significantly inhibited by aqueous extracts from BOT. This finding indicated that the decreased P-gp transport capacity was related to the inhibition of MDR1 mRNA expression.

### 3.8. Effects of FBOT and HBOT aqueous extracts on PAH concentration In renal sections, renal OAT1 and OAT3 mRNA expressions in mice

**Figure 6:**
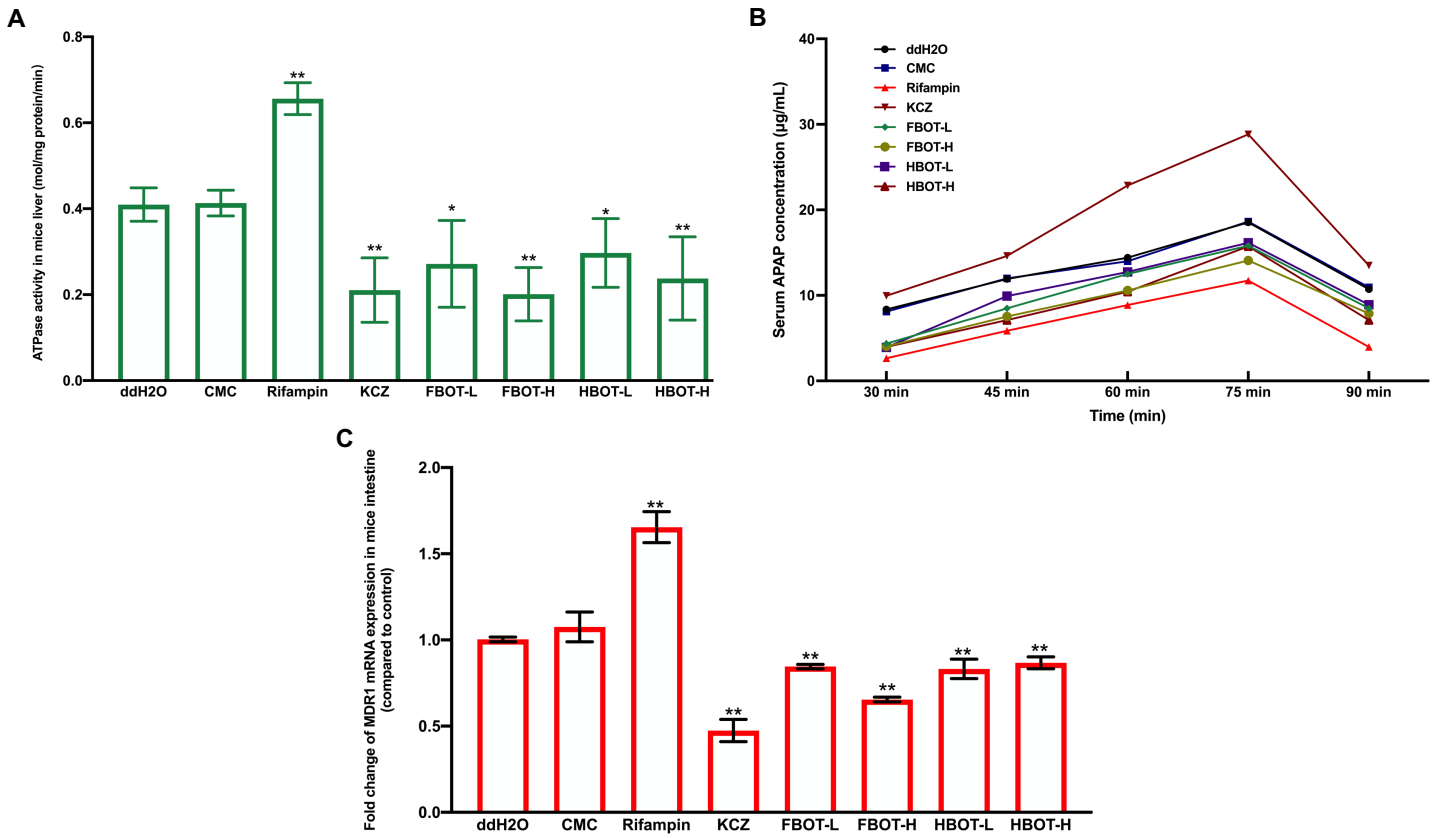
The PAH concentration on mice renal slides **(A)**, OAT1 **(B)** and OAT3 mRNA expressions **(C)** in mice kidney. Values are mean ± SD (*n* = 3). Bars with different letters represent a significant difference between each group (*p* < 0.05). HBOT-L and HBOT-H mean low and high dose of hot air-drying Baiyedancong Oolong tea, respectively.

PAH can be transported almost completely by renal OATs at low concentration. In this study, the amount of PAH uptake in kidney sections was measured in the way of *in vitro* incubation to confirm the effect of aqueous extracts from BOT on OATs transport ability. The results are indicated in Figure 6A.

There was no significant difference in the concentration of PAH between the control and the CMC group (*p* > 0.05), suggesting that CMC had no significant effect on the ability of OATs to ingest PAH. Compared with the control group, the concentration of PAH in kidney sections was significantly reduced by probenecid (*p* < 0.05), a potent inhibitor of transporter OATs, which was only 16% of the control group. Similarly, aqueous extracts of BOT significantly reduced the concentration of PAH in kidney sections in a dose-dependent manner. High and low dose of BOT treatment were 25, 32, 23 and 28% of the control group, respectively. In addition, no significant difference was obtained in the PAH concentration between FBOT-H and HBOT-H, while there was obvious difference between FBOT-L and HBOT-L. This difference might be resulted from their different chemical compositions. However, this still needs to be further studied. Renal OATs play an important role in the elimination of organic anions from the body, including uric acid. Herein, these observations suggest that aqueous extracts from BOT could inhibit the OATs transport ability, thereby reducing the renal excretion of substrate drugs or drug metabolites.

The mRNA expressions of renal OAT1 and OAT3 in mice was determined by RT-PCR assay to explore whether the decreased OATs transport capacity is caused by decreased transcription of OAT1 and OAT3. The results are depicted in Figures 6B,C, respectively. Generally, similar patterns were found in OAT1 and OAT3 mRNA levels with the PAH concentration. Probenecid and aqueous extracts from BOT had significant inhibitory effects on OAT1 and OAT3 mRNA expressions. Combined with OAT1 and OAT3 mRNA expressions and transport capacity analysis, aqueous extracts from BOT provided consistent downregulation of OAT1 and OAT3 mRNA and its corresponding transport capacity. The results suggested that the decreased OATS transport capacity was strongly related to the downregulation of Oat1 and OAT3 mRNA expression.

## 4. Discussion

Tea is one of the three non-alcoholic beverages in the world. The consumption of a large number of tea health care products and medicines has greatly improved the correlation between tea and our Daily life. With in-depth studies on drug interactions, most drug interactions are due to changes in P450 activity or/and transporter-mediated drug metabolism level *in vivo*, which ultimately lead to reduced efficacy or increased toxicity (20). Due to the health benefits and its medicinal value of green tea, more studies have been conducted on its influence on drug metabolism and its potential mechanisms, among which the most studies have been focused on CYP3A enzymes (21). Metabolic drug interactions caused by induction and inhibition of P450 enzymes may significantly change the pharmacokinetics, efficacy, and toxicity of combined drugs. Hence, elucidating the induction and inhibition of P450 is an essential part for drug safety and efficacy evaluation (22). The abilities of CYP3A11, CYP2E1 and CYP2C37 isoenzymes of P450 in mice are corresponding to CYP3A4, CYP2E1 and CYP2C9 in human body (23). However, the results are not consistent either *in vivo* or *in vitro* studies, and further exploration is needed. Therefore, this study investigated the effects of aqueous extracts from BOT on mice small intestine, liver, and kidney, involving multiple links of drug absorption, metabolism and excretion. Herein, mice were administrated with high and low doses of FBOT (1463.7 and 292.74 mg/kg/d, respectively) and HBOT (1454.46 and 290.89 mg/kg/d, respectively) twice a day for 7 consecutive days. Our results indicated that both high and low doses of FBOT and HBOT aqueous extracts could significantly increase LM CYP3A, CYP2E1 and CYP2C37 activities and IM CYP3A activity. Previous studies have shown that tea polyphenols induced the expressions of CYP1A1, CYP1A2, CYP2D6, CYP2E and CYP3A4 as well as improved their activities (24, 25). However, contrasting observations have emerged. One pre-clinical study from Chow et al. (26) revealed that green tea polyphenols regulated the activity of P450 system. However, their subsequent clinical experiments showed that the activity of P450 enzyme varied greatly in healthy volunteers after administrated with tea polyphenols containing 800 mg of EGCG before a meal every day for 4 weeks. The intervention of green tea polyphenols for 4 weeks may have a weak inhibitory effect on the activity of CYP3A4, while there was no obvious change in CYP1A2, CYP2D6 and CYP2C9 activities (26). This might be related to the difference in such conditions as the usage doses, the cell line used, and the duration of action during the experiments. A significant difference was observed in this study in the induction of enzyme activities between high and low dose of BOT aqueous extracts, among which the efficiency of FBOT-H and HBOT-H was similar to that of rifampicin, which is a powerful inducer for CYP3A activity. This suggest that the aqueous extracts from BOT-H significantly could influence the metabolism of CYP3A substrate drugs in the liver, thereby increasing the metabolism and reducing the drug efficacy. This observation should be taken seriously in clinic. Therefore, aqueous extracts of BOT may significantly improve the liver metabolic efficiency of CYP3A4, CYP2E1 and CYP2C9 to a large number of substrate drugs, thereby improving the liver first-pass effect of substrate drugs and reducing the biological effectiveness of drugs.

Previous studies have indicated that tea polyphenols regulate enzyme activities by inducing gene expressions of CYP1A1, CYP1A2, CYP2D6, CYP2E1 and CYP3A4 (27). Park et al. (28) found that green tea extracts reduced CYP3A gene expression, while increased CYP2B gene expression in rats. The results of previous studies are not consistent. Herein, the results showed that the increased mRNA expressions of liver CYP3A11, CYP2C37 and small intestine CYP3A11 were consistent with the increased corresponding enzyme activities, and the upregulation of transcription level increased the enzyme activity. Combined with the liver CYP3A enzyme activity and mRNA expression. In addition, the liver CYP3A enzyme activity in mice treated with BOT-H was similar to that treated with rifampicin, while its mRNA expression was significantly lower than that of rifampicin, suggesting that apart from induction of mRNA expression of CYP3A11, aqueous extracts from BOT may improve its enzyme activity through other ways. Nuclear receptors are widely distributed in the body and can affect the absorption, distribution and excretion of drugs and their metabolites by regulating gene expressions of a variety of drug metabolism enzymes and transporters (29). PXR, CAR and PPAR regulate the expression of the CYP3A expression (30). CYP3A inducers activate PXR to induce the expression of CYP3A and other isoforms of P450 *via* DR3, ER6 and IR6 (31). This pathway needs to be further studied in the future study. However, aqueous extracts from BOT significantly inhibited the expression of CYP2E1 mRNA, which was not agreement with its enzyme activity. This might be due to the downregulation of the transcription level of CYP2E1, which reduced its protein expression and thus exhibited a negative regulatory effect. Previous studies have shown that alcohol induced increased CYP2E1 activity was mainly regulated by increasing the stability of post-transcriptional proteins (32). PPAR is involved in the regulation of CYP2E1 gene expression (33). However, it still needs to be further verified if the increased CYP2E1 activity is regulated by either PPAR or improving the protein stability.

P-gp, which can reverse pump substrate drugs out of cells, is widely found in the intestinal tract. It has a wide range of drug substrate specificity and can participate in the transport processes of drug absorption, distribution and excretion in the body, playing an important role in pharmacokinetics and pharmacodynamics (34). Aqueous extracts from BOT decreased the activity of ATPase in small intestine and increased the serum concentration of APAP of mice. At 75 min, the peak values of serum APAP concentration in high and low doses of FBOT and HBOT were 22.69, 18.30, 22.22 and 17.17 μg/ml, respectively, which were significantly higher than those in the control group at the same time point. In this study, ATPase activity and the serum APAP accumulative concentration were used to evaluate the transport capacity of P-gp. The results showed that the ability of small intestinal P-gp to discharge the substrate drug into the intestinal lumen was reduced, leading to the reduced absorption of the substrate drug in the small intestine. This is consistent with previous results. However, Jodoin et al. (35) studied the effects of black and green tea that are commonly seen in the market, finding that drinking green tea for consecutive 7 days significantly inhibited the intestinal transporter P-gp, while drinking black tea induced the intestinal P-gp. It still needs to be verified in future studies whether aqueous extract from BOT can regulate P-gp transport capacity in other tissues and organs. Our results showed that aqueous extracts from BOT significantly inhibited the transcription level of MDR1 in the small intestine of mice, suggesting that the weakened intestinal P-gp transport capacity was likely related to the MDR1 transcription level and the decreased ATPase activity. This was agreement with the results from Maleki Dana et al. (36), finding that KB-A1 cells treated with 40 μg/ml tea polyphenols or 10 μg/ml EGCG inhibited P-gp activity by suppressing its ATPase activity. Some studies have also shown that green tea polyphenols or green tea extracts could reduce the transport capacity of P-gp by inhibiting the P-gp gene expression (37, 38).

OATs, a member of the solute carrier (SLC) superfamily, is an ingestion carrier, which can transport substrates into cells. It is mainly distributed in the kidney proximal convoluted tubules and plays an important role in the excretion of drugs and their metabolites in the body (39). OATs can mediate the entry of organic anion compounds (including drugs and their metabolites) from the extracellular fluid or blood into the renal tubule lumen epithelial cells, which are then secreted into the renal tubule by other efflux transporters and excreted in the urine (40). Currently, the drug interaction mediated by OATs between western and traditional Chinese medicine has become one of the hot spots in pharmacokinetics studies, of particular interested on the study of drug nephrotoxicity mediated by reduced OATs transport ability. However, no study, if any, focused on the effect of tea polyphenols on OATs or the effects of BOT on drug metabolizing enzymes and transporters till now. Our results revealed that both FBOT and HBOT could significantly inhibit the transport capacity of OATs in kidney. It was found that high and low doses of BOT greatly reduced the concentration of the typical substrate PAH of OATs in kidney sections, and this effect was similar to the that of probenecid. Reduced drug excretion rate leads to the increased blood drug concentration as well as enhanced the drug efficacy. This may have side effects or even toxicosis. Therefore, the effect of BOT on reducing OATs transport capacity needs to cause our values. Furthermore, our findings revealed that renal OAT1 and OAT3 transcription levels were significantly reduced by aqueous extracts from BOT, which was consistent with the decreased uptake capacity of substrate PAH by OATs, implying that BOT extract could simultaneously reduce the transcription of renal OAT1 and OAT3, thereby reducing the transport and excretion of organic anions and drug metabolites. Besides, it was observed that at low dose level, the uptake ability of OATs in mice to PAH was significantly different between FBOT-L and HBOT-L. This could be due to the different contents of flavonoids between FBOT and HBOT. The renal handling of the flavonoid and its effect on renal transporters is a vital issue from the standpoint of nutritional research. Interactions with various dietary components, such as flavonoids (e.g., EGCG) and their conjugates, phenolic acids, and phenylpropanoids, are demonstrated by inhibiting OAT-mediated transport of model substrates (41). Our preliminary study has shown that compared with traditional hot drying technique, freeze-drying technique could retain more tea polyphenols and amino acids (the data did not show). Hence, it could result in an improved PAH uptake ability. However, this observation needs to be further explored by identifying the specific polyphenol compositions in FBOT and HBOT.

## 5. Conclusion

Collectively, aqueous extracts from FBOT and HBOT could improve the small intestinal and liver CYP3A, liver CYP2E1 and CYP2C37 activities in mice, while decrease the transport capacity of intestinal effluent transporter P-gp and renal ingestion transporter OATs. Thus, it comprehensively affected the metabolisms of oral drugs in liver, absorption in intestine, and excretion in renal. This study was the first step to investigate the effects of BOT on drugs. So far, at least various aspects need further exploration. For instance, BOT is rich in theanine, catechin monomers and tea pigments. These bioactive compounds may exhibit the regulatory effects on enzyme activities of liver CYP3A, CYP2E1 and CYP2C37. Additionally, neither animal nor cell experiments can replace clinical trials, which would be conducted in our future research to verify the influence of tea on drug effects through clinical trials.

## Data availability statement

The original contributions presented in the study are included in the article/supplementary material, further inquiries can be directed to the corresponding author/s.

## Ethics statement

The animal study was reviewed and approved by the Laboratory Animal Center of Guangzhou University of Traditional Chinese Medicine.

## Author contributions

MZ contributed to conception and design of the study, organized the database, performed the statistical analysis, and wrote the first draft of the manuscript. ZQ wrote sections of the manuscript. All authors contributed to manuscript revision, read, and approved the submitted version.

## Funding

This study was kindly supported by Study on Quality Improvement of High Mountain Organic Tea in Xinfeng County (D11920716); Introduction and Transformation of Tea Invention Patent (D11920714); Yunfu local tea population species Resource utilization and Production technology Integration Demonstration (KA22Y0148) and Lianjiang Characteristic granular oolong tea new product development and technology integration demonstration (KA22Y0125).

## Conflict of interest

The authors declare that the research was conducted in the absence of any commercial or financial relationships that could be construed as a potential conflict of interest.

## Publisher’s note

All claims expressed in this article are solely those of the authors and do not necessarily represent those of their affiliated organizations, or those of the publisher, the editors and the reviewers. Any product that may be evaluated in this article, or claim that may be made by its manufacturer, is not guaranteed or endorsed by the publisher.
