# Supplementary material for: Effects of aqueous extract from Baiyedancong-Oolong tea on cytochrome P450 enzymes activities, P-gp and OATs transport abilities and transcription levels in mice
Source: Front Nutr. 2023 May 9;10:1136329. doi: 10.3389/fnut.2023.1136329 (PMC10205018; doi:10.3389/fnut.2023.1136329)
Supplement: Supplementary file 1 [file Table_1.docx]

| **Table 1. Prime sequences for RT-PCR** | | |
| --- | --- | --- |
| **Gene** | **Forward prime(5’ →3’)** | **Reverse prime(5’ →3’)** |
| **CYP3A11** | CACAAACAAGCAGGGATGGAC | GGTAGAGGAGCACCAAGCTG |
| **CYP2E1** | CACCGTTGCCTTGCTTGTCTG | CTCATGAGCTCCAGACACTTC |
| **CYP2C37** | CTGCATGACAGCACGGAGTT | GTGGCCAGGGTCAAATTTCTC |
| **MDR1** | CCCATCATTGCAATAGCAGG | GTTCAAACTTCTGCTCCTGA |
| **OAT1** | ATGCCTATCCACACCCGTGC | GGCAAAGCTAGTGGCAAACC |
| **OAT3** | CAGTCTTCCTGGCAGGTATA | CTGTAGCCAGCGCCACTGAG |
| **GAPDH** | GGTGAAGGTCGGTGTGAACG | CTCGCTCCTGGAAGATGGTG- |
